# Thermostable vacuum foam dried Newcastle disease vaccine: Process optimization and pilot-scale study

**DOI:** 10.1007/s00253-024-13174-7

**Published:** 2024-06-05

**Authors:** Fang Lyu, Yan-hong Zhao, Xiao-xin Zuo, Babalwa Nyide, Bi-hua Deng, Ming-xu Zhou, Jibo Hou, Jia-jie Jiao, Min-qian Zeng, Hong-ying Jie, Ademola Olaniran, Yu Lu, Thandeka Khoza

**Affiliations:** 1 Institute of Veterinary Immunology & Engineering, National Research Center of Engineering and Technology for Veterinary Biologicals, Jiangsu Academy of Agricultural Sciences, Nanjing, 210014 China; 2 Department of Biochemistry, School of Life Sciences, College of Agriculture, Engineering & Science, University of KwaZulu-Natal, Pietermaritzburg, 3209 South Africa; 3 Department of Microbiology, School of Life Sciences, Engineering & Science, College of Agriculture, University of KwaZulu-Natal, Durban, 4000 South Africa; 4 GuoTai (Taizhou) Center of Technology Innovation for Veterinary Biologicals, Taizhou, 225300 China; 5 School of Animal Medicine, Nanjing Agricultural University, Nanjing, 210095 Jiangsu China; 6 Jiangsu Key Laboratory for Food Quality and Safety-State Key Laboratory Cultivation Base, Ministry of Science and Technology, Nanjing, 210014 China; 7 School of Pharmacy, Jiangsu University, Zhenjiang, 212013 China

**Keywords:** Vacuum foam drying, Process upscaling, NDV vaccine, Thermostability

## Abstract

**Abstract:**

Vacuum foam drying (VFD) has been shown to improve the thermostability and long-term shelf life of Newcastle Disease Virus (NDV). This study optimized the VFD process to improve the shelf life of NDV at laboratory-scale and then tested the optimized conditions at pilot-scale. The optimal NDV to T5 formulation ratio was determined to be 1:1 or 3:2. Using the 1:1 virus to formulation ratio, the optimal filling volumes were determined to be 13–17% of the vial capacity. The optimized VFD process conditions were determined to be at a shelf temperature of 25℃ with a minimum overall drying time of 44 h. The vaccine samples prepared using these optimized conditions at laboratory-scale exhibited virus titer losses of ≤ 1.0 log_10_ with residual moisture content (RMC) below 3%. Furthermore, these samples were transported for 97 days around China at ambient temperature without significant titer loss, thus demonstrating the thermostability of the NDV-VFD vaccine. Pilot-scale testing of the NDV-VFD vaccine at optimized conditions showed promising results for up-scaling the process as the RMC was below 3%. However, the virus titer loss was slightly above 1.0 log_10_ (approximately 1.1 log_10_). Therefore, the NDV-VFD process requires further optimization at pilot scale to obtain a titer loss of ≤ 1.0 log_10_. Results from this study provide important guidance for possible industrialization of NDV-VFD vaccine in the future.

**Key points:**

*• The process optimization and scale-up test of thermostable NDV vaccine prepared through VFD is reported for the first time in this study.*

*• The live attenuated NDV-VFD vaccine maintained thermostability for 97 days during long distance transportation in summer without cold chain conditions.*

*• The optimized NDV-VFD vaccine preparations evaluated at pilot-scale maintained acceptable levels of infectivity after preservation at 37℃ for 90 days, which demonstrated the feasibility of the vaccine for industrialization.*

**Supplementary Information:**

The online version contains supplementary material available at 10.1007/s00253-024-13174-7.

## Introduction

Newcastle disease (ND) caused by avian paramyxovirus is one of the poultry diseases that causes great economic losses to poultry farmers worldwide (Ross et al. 2022; Shang et al. 2022). Vaccination is an effective measure for the prevention and control of this disease, however the currently available commercial live attenuated vaccines for ND are dependent on strict adherence to cold chain to maintain vaccine efficacy (Pambudi et al. 2022; Singh et al. 2022). As a result, alternative innovative preservation strategies are continuously explored to mitigate the challenges of vaccine cold chain reliance (Crommelin et al. 2021; Leung et al. 2019). Previous small-scale studies have demonstrated that vacuum foam drying (VFD) improves the thermostability and long-term shelf life of the Newcastle disease virus (NDV) vaccine while maintaining its immunogenicity profile (Lyu et al. 2022). This signifies a breakthrough in the development of cold-chain independent vaccines (Fanelli et al. 2022; Lyu et al. 2022). However, the outcomes of small-scale bioprocesses do not always provide a reliable simulation of their large-scale equivalents due to differences in the physico-chemical dynamics of micro- and macro-environments that affect biological systems (Garcia-Ochoa and Gomez 2009). As such, the feasibility of large-scale production of the NDV-VFD vaccine is yet to be validated.

Prior to registration and licensing of vaccines for commercialization, vaccine research undergoes three major phases, namely (i) research and development (R&D), (ii) process optimization and scale-up, and (iii) clinical trials (Kanesa-thasan et al. 2011). The R&D phase involves a small-scale investigation into the feasibility of the vaccine design and formulation to establish the efficacy and safety of the vaccine in laboratory studies. In contrast, the process optimization and scale-up phase is defined as “the consolidation of a large-scale process based on results obtained from experiments with small-scale equipment” (Bastos et al. 2015). This phase involves determination of the optimal bioprocess parameters for efficient large-scale vaccine production while maintaining product quality, safety and stability. As a quality assurance standard for licensure, the optimized bioprocess must yield a reproducible product in a manufacturing environment. Lastly, the clinical trials phase involves establishing the product safety and quality (Kanesa-thasan et al. 2011). Vaccine safety, quality and efficacy, however, are largely affected by the complexities of scaling up vaccine production from laboratory scale to pilot scale.

Like any new product manufacturing system, commercial vaccine production must be scalable for large-scale production and supply to meet global demand (Smith et al. 2011). However, the greatest hurdle for commercial vaccine manufacturers to overcome is the challenge of realizing an industrial vaccine product that elicits an immunological response similar to that observed during the smaller laboratory scale study (Bastos et al. 2015). Notably, small-scale preparation of biologics and vaccines in the laboratory is typically achieved in smaller quantities in a micro-environment with controlled parameters (Bastos et al. 2015; Damle et al. 2020; Garcia-Ochoa and Gomez 2009; Kanesa-thasan et al. 2011; Smith et al. 2011). In contrast, industrial-scale production of biologics is typically conducted in large equipment (a macro-environment), which typically offers less control over batch homogeneity (Wang et al. 2015). For example, in large bioreactors (e.g. 100 L / 150 L), critical parameters such as dissolved oxygen, nutrient availability, pH and mixing rates vary greatly compared to laboratory shake-flasks, which affects cell metabolism and growth rates. Hence, laboratory-scale procedures often require readjustment prior to application for large-scale production, which can negatively affect the quality, safety and efficacy of biological products (Pisano et al. 2013b; Plotkin et al. 2017). Similarly, the application of lyophilization in the VFD process from laboratory-scale to pilot-scale introduces several parameter changes such as increased cabin volume and shelf area. By association, these changes can have undesired implications for other process variables such as heating and cooling rates, shelf temperature, vapor pressure accuracy, and water capture efficiency (Assegehegn et al. 2020; Butreddy et al. 2020). As such, upscaling production from small-scale to large-scale requires process optimization to mitigate technical challenges that cause poor product quality and batch inconsistency on the production line, which have severe financial ramifications for commercial vaccine manufacturing (Butreddy et al. 2020; Plotkin et al. 2017; Tortora et al. 2010).

Vaccine thermostability has a direct bearing on the shelf life and safety of the commercial product; therefore, it is important to consider the relationship between vaccine preservation methods and the feasibility of upscaling vaccine production (Bastos et al. 2015; Butreddy et al. 2020). Our previous study made a strong case for the use of VFD technology for the production of a robust, thermostable and immunogenic NDV vaccine (Lyu et al. 2022). Likewise, there is more published literature to support the successful long-term stabilization of sensitive biological material using the VFD technique (Bronshtein et al. 2001; Tlaxca et al. 2015). Particularly interesting is that there is consensus among various literature sources in support of the scalability of the VFD technique, which was revolutionized by Bronshtein et al. (Bronshtein et al. 2001) in 2001 through a patented industrial-scale barrier technology invention (Bronshtein et al. 2001; Doekhie 2019; Pisal et al. 2006a, b; Tlaxca et al. 2015). However, the main limitation of this technology is that to prevent sample overflow during the VFD process, the working capacity of the foaming chamber should not exceed 25% of the total chamber capacity in order to accommodate sample expansion caused by foaming (Bronshtein et al. 2001; Doekhie 2019; Pisal et al. 2006a, b; Tlaxca et al. 2015). This may have significant financial implications for vaccine manufacturers, who seek to maximize turnover rates while minimizing cost-to-output ratios. Other important considerations with VFD include temperature and pressure control, bubble nucleation and growth rates, liquid viscosity, and foaming chamber geometry (Tlaxca et al. 2015).

Lastly, Tlaxca et al. (Tlaxca et al. 2015) emphasize that, although the application of VFD can improve vaccine stability, this advantage does not automatically apply universally. Thus, drawing on the findings of our previous study, we next sought to address the question of upscaling VFD of the NDV vaccine. Hence, this study aims to optimize the NDV-VFD process at laboratory scale and further test the optimized condition at pilot scale. The findings from this study will provide necessary guidance for the future adoption of this process by the vaccine manufacturing industries.

## Materials and methods

### Materials

Live attenuated NDV (*LaSota* strain) was obtained from the National Institute of Veterinary Drug Control (Beijing, China). The specific pathogen-free (SPF) chicken eggs were purchased from Beijing Boehringer Ingelheim Viton Biotechnology Co., Ltd. All the reagents were purchased from Sigma-Aldrich (St. Louis, USA). The vials were purchased from Schott Glass Technology Co., Ltd. (Suzhou, Germany). Brombutyl rubber stoppers (Siliconized, Datwyler, Schattdorf, Switzerland) were used in this study.

The following Lab Lyophilizers sizes were used in this study: 0.27 m^2^ (SP Scientific, Warminster, VirTis Advantage Pro, PA, USA), and 0.5 m^2^ (Tofflon, LYO-0.5, CHN). For pilot-scale studies, Pilot Lyophilizers of 7.5 m^2^ (Tofflon, LYO-7.5, CHN) and 15 m^2^ (Tofflon, LYO-15, CHN) were used.

### Process optimization for VFD using the Lab Lyophilizer

This study is a follow up of our previous work where we demonstrated that NDV vaccine formulated in T5 and dried using VFD method exhibited improved thermostability without compromising efficacy (Lyu et al. 2022). In the present study, we sought to optimize the VFD process for potential application at pilot scale. Thus, the VFD process was optimized by determining the optimal conditions for (a) ratio of virus to T5 formulation, (b) vaccine liquid loading volume, (c) shelf temperature, and (d) overall drying time during the VFD process. Additionally, the effect of sample positioning in the lyophilizer on vaccine efficacy was determined. To achieve this, we tested the homogeneity of the vaccine samples located on different shelves and positions of the lyophilizer during VFD by assaying virus titration, residual moisture content (RMC), and the stability of the samples.

NDV was propagated by inoculation into the allantoic cavity of 9–11-day-old embryonated SPF eggs and incubated at 37℃ for 120 h. The allantoic fluid was then harvested and stored at -20℃ for further use. The harvested fluid was formulated with T5 formulation [15% trehalose, 2.5% gelatin, 0.05% pluronic F127, and 25 mmol/L potassium phosphate buffer (pH 7.0–7.4)] at various ratios to prepare the vaccine samples. Samples were then vacuum foam dried using a LabLyo1 laboratory lyophilizer (SP Scientific, Warminster, PA, USA, 0.27 m^2^) as described by Lyu et al (2022).

The virus titer was detected using the egg infectious dose 50 (EID_50_) method as described by Reed and Muench (1938). Briefly, the VFD vaccines were reconstituted in phosphate buffered saline (pH 7.4) and diluted serially (tenfold), resulting in 10^–6^ to 10^–9^ dilutions of the virus. One hundred microliters of each dilution were injected into the allantoic cavity of 9–11-day-old SPF embryonated egg for a total of 5 eggs per dilution. The eggs were incubated at 37℃ for 120 h, and the embryos found dead were discarded. Allantoic fluid from the eggs was then harvested and analyzed using the hemagglutination assay (HA). The EID_50_ titer of the NDV, as determined by the number of HA-positive and HA-negative eggs in each dilution, was calculated using the Reed and Muench method in triplicate.

The RMC of the NDV-VFD vaccine was detected using thermogravimetric analysis (Merivaara et al. 2021; Osman et al. 2021; Regis et al. 2021). To determine the RMC after drying, eight vials of each vaccine preparation were weighed before and after drying at 80℃ for 2 h without a stopper. Thereafter, the RM % was calculated as follows: RM% = [(Initial weight—Final constant weight)/Initial weight] × 100. Samples with a virus titer loss of ≤ 1.0 log_10_ EID_50_/mL and a RM% ≤ 3% were considered to be of an acceptable standard. Further to this, the foaming rate (FR) of the samples was calculated using the following equation: FR% = (No. of successfully foamed vials/Total no. of vials) × 100 (Lv et al. 2016; Zuo et al. 2020). Lastly, the accelerated stability (AS) test was used to determine the stability of the NDV-VFD vaccine under elevated stress conditions. To achieve this, vaccine preparations were incubated at 45℃ for 7 days and virus titer levels (EID_50_) were determined before and after incubation to determine the virus titer losses.

### Optimization of virus to T5 formulation ratio

In the process of vaccine industrialization, the vaccine ratio is typically adjusted according to the vaccine production specifications. During the VFD process, however, different ratios of virus to formulation may negatively affect foam formation, thus it is important to optimize this process parameter for VFD vaccines. Therefore, six ratios (1:1, 1:2, 1:3, 2:1, 3:1 and 3:2) of NDV to T5 formulation were evaluated. Factors considered in identifying the optimal ratio of virus to T5 formulation were foam formation, foam overflow, RMC, and vaccine thermostability using the AS test.

### Optimization of vaccine liquid loading volume

Another critical factor that affects foam formation during the VFD process is the vaccine liquid volume. Therefore, the filling capacity of three different sizes of vials (7 mL, 10 mL and 30 mL) was tested. Briefly, five 7 mL vials and five 10 mL vials were filled with different volumes (i.e. 0.8 mL, 1.0 mL, 1.2 mL, 1.5 mL and 2.0 mL) of vaccine preparation consisting of NDV and T5 formulation in a 1:1 ratio. Similarly, 30 mL vials were filled with 2.0 mL, 3.0 mL, 4.0 mL and 5.0 mL of this NDV-T5 formulation. After the VFD process, foam overflow, RMC, and AS test were conducted to determine the optimized loading volume.

### Optimization of shelf temperature during the VFD process

The laboratory VFD process was performed as described by Lyu et al. (2022). This process consisted of four steps in the following sequence: (a) The test sample was placed into a lyophilizer (SP Scientific, Warminster, PA, USA, 0.27 m^2^) and kept for 30 min after the shelf temperature reached 25℃; (b);the pressure of the dryer chamber was decreased in a stepwise manner from 600 mBar (60 × 10^3^ Pa) to 3 mBar (300 Pa) over approximately one hour at a set temperature (15℃, 20℃, 25℃, and 35℃) to identify the optimal shelf-temperature for the VFD process; thereafter, the temperature was re-adjusted to 25℃ and maintained for 2 h at a pressure of 3 mBar (300 Pa); (c) the vacuum was decreased to 0.1 mBar (10 Pa) over 12 h and (d) finally, the chamber pressure was decreased to 0.01 mBar (1.0 Pa) for 32.5 h to make the overall drying time for the VFD process 48 h in length. Four shelf temperature conditions were tested to identify the optimal shelf temperature for the VFD process. The other steps were kept constant while varying the shelf temperature.

### Optimization of overall drying time during the VFD process

Following identification of the optimal shelf temperature, the overall drying time of the VFD process for the NDV-VFD vaccine preparation was optimized. Briefly, the VFD process was performed as described in steps (a)—(c) in 2.2.3 using the optimal shelf temperature. In step (d) the pressure was decreased to 0.01 mBar (1.0 Pa) and held for variable times (2.5, 14.5, 20.5, 24.5, 28.5 and 32.5 h) to determine the optimal drying duration of the VFD process. Therefore, the total VFD drying time varied between 18 and 48 h. Samples were taken after each VFD process and analyzed for NDV titer loss and RMC.

### Investigating the effect of sample positioning during the VFD process

Given that samples in the LabLyo1 lyophilizer are located in different positions across three shelves, we then determined if the positioning of the samples has any effect on the RMC and virus titer loss (EID_50_/mL) following VFD. Briefly, NDV and T5 formulation were mixed in a ratio of 1:1 and dispensed as 1.0 mL aliquots into 10.0 mL vials. A total of 429 vials were loaded on 3 shelves. The samples were then vacuum foam dried using the optimized conditions (as determined in the aforementioned experiments) for VFD process. Figure S1 shows the locations of the samples that were selected for analysis based on the shelf number (1 – 3) and position (A – I). Positions A, C, G and I represent samples at the 4 corners of each shelf. Positions B, D, H and F represent samples at the 4 edges of each shelf, and position E represents samples at the center of each shelf. Eleven vials of VFD vaccine were randomly selected from each position on the shelves; three vials were used for the virus titer test and 8 vials for the RMC detection.

### NDV-VFD vaccine stability after long-distance transportation without cold chain

To evaluate the long-term stability of NDV-VFD vaccines when transported without any cooling measures, NDV-VFD vaccine samples was prepared using the optimized process and conditions identified above. NDV vaccine samples prepared through freeze-drying (FD), which is prepared as described in our previous study (Lyu et al. 2022), were used as a control. One hundred vials each of NDV-VFD and NDV-FD samples were packed together in a cardboard box, and then transported around China for 97 days during summer from June to August. Transportation commenced from (1) Nanjing in Eastern China, to (2) Chifeng in Northern China, to (3) Zunyi in Southwest China, to (4) Shenzhen in South, before returning to the starting point. During transportation, the samples remained in Chifeng, Zunyi and Shenzhen for 21 days each at ambient temperature, and the average journey time between stations ranged from 7–9 days. The overall duration for this experiment was 97 days, during which the ambient temperature and humidity of the environment were monitored daily. The virus titers of the vaccine samples before and after transportation were detected using the egg infectious dose 50 (EID_50_) method (Reed and Muench. 1938). Three vials of each sample were randomly selected for detection.

### Upscaling the VFD process from lab-scale to pilot-scale

To study the feasibility of VFD in pilot-scale lyophilizers, the optimized drying cycle was integrated into the NDV-VFD process on different scales from lab to pilot lyophilizers. The specific information of these lyophilizers is shown in Table 1. The operational parameters for the VFD process in each lyophilizer specification were recorded, including shelf and product temperature, condenser temperature and vacuum pressure. To evaluate the long-term stability of NDV-VFD vaccine preparations made using lab-scale and pilot-scale lyophililizers, the samples were evaluated using the AS test (45℃ for 7 days), RM% and FR% analysis. The conditions that met the selection criteria (i.e. virus titer losses of ≤ 1.0 log_10_, RM% ≤ 3%, and FR% ≥ 90%) were used to prepare samples for further testing by storing these samples at 37℃ for 90 days.

**Table 1:** Specifications for Lab and Pilot lyophilizers used in this study

Lyophilizer LabLyo/ PilotLyo	Manufacturer	Model	No. of shelves	Shelf area (m^2^)	Load capacity (10 mL Vials)	Lowest condenser temperature (℃)	Condenser capacity (L)
LabLyo1	SP Scientific	VirTis Advantage Pro	3	0.27	429	-67	6 L
LabLyo2	Tofflon	LYO-0.5	4	0.54	896	-75	8 L
PilotLyo1	Tofflon	LYO-7.5	7	7.5	10,710	-75	150 L
PilotLyo2	Tofflon	LYO-15	7	15	22,950	-75	300 L

### Statistical analysis

All of the statistical analysis were performed by GraphPad Software Prism 6.0 (GraphPad Software Inc., San Diego, CA, USA). The data were presented as mean ± standard deviation or n (%). Statistical differences between experimental groups were determined using the analysis of variance method. Statistical significance was set as follows: *, p < 0.05; **, p < 0.01; ***, p < 0.005; ns, not significant.

## Results

### Determination of the optimum volume ratio of virus toT5 formulation

In our previous study, vacuum foam dried vaccine preparations comprising a 1:1 ratio of NDV and T5 formulation exhibited acceptable thermostability and efficacy, but no other ratios were tested (Lyu et al. 2022). Therefore, subsequent to that study, we sought to determine if different virus to T5 formulation ratios (i.e. 1:2, 1:3, 2:1, 3:1 and 3:2) would have any significant impact on foam formation and overflow during the VFD process. As shown in Table 2, no foaming was observed in samples consisting of ratios of 1:2 and 1:3 of virus to T5 formulation. In contrast, foaming was observed in samples consisting of 1:1, 2:1, 3:1 and 3:2 of virus to T5 formulation. Despite observing no foam formation in samples with 1:2 and 1:3 ratios, samples prepared at both of these ratios overflowed during the VFD process, indicating excessive bubbling. Therefore, samples that either showed no foam formation, or foamed but overflowed during the VFD process were not considered for further validation using residual moisture content (RMC) and virus titer loss analyses. Subsequently, samples prepared with 1:1 and 3:2 volume ratios of virus to T5 formulation were further assayed using the RMC analysis and the AS test. The 1:1 and the 3:2 samples showed acceptable virus titer losses of 0.73 ± 0.14 log_10_/mL and 0.87 ± 0.10 log_10_/mL, respectively after storage at 45℃ for 7 days, with both samples exhibiting RM values of < 3%. Therefore, virus to T5 formulation ratios of 1:1 or 3:2 were suitable for NDV-VFD vaccine preparations. Furthermore, samples prepared with the ratio of 1:1 were used in subsequent studies, since this ratio showed lower virus titer losses during the AS test with lower RMC. Only vaccines that foamed and exhibited no overflow (i.e. 1:1 and 3:2 ratios) were further evaluated using the AS test and RM analysis.

**Table 2:** The appearance and overflow phenomenon at different virus to T5 formulation ratios (V_virus_:V_formulation_)

Volume ratio of virus to T5 formulation (V_virus_:V_formulation_)	Overflow phenomenon ( ±)	Appearance	NDV titer (log EID_50_/mL) (n = 3)		NDV titer loss after storage at 45 ℃ for 7 days (log EID_50_/mL)	RM%
			Before storage	After storage at 45℃ for 7 days		
1:1	-	Foamed	8.56 ± 0.17	7.83 ± 0.11	0.73 ± 0.14	2.05 ± 0.21
1:2	-	Dried with no foam	UD	UD	UD	UD
1:3	-	Dried with no foam	UD	UD	UD	UD
2:1	+	Foamed	UD	UD	UD	UD
3:1	+	Foamed	UD	UD	UD	UD
3:2	-	Foamed	8.75 ± 0.15	7.88 ± 0.13	0.87 ± 0.10	2.35 ± 0.19

 + : Overflow observed; -: No overflow observed. UD: Undetected. Seventy vials were prepared at each ratio of virus: T5 formulation.

### Optimal fill volume in vials with different sizes

In order to accommodate sample expansion caused by foam formation during the VFD process, the vial size and filling volume must be carefully considered. With this in mind and the possibility for scale-up applications, we investigated the maximum acceptable filling volume for different vial sizes. NDV and T5 formulation were mixed in equal volumes, and then filled into 7 ml, 10 mL and 30 mL vials with different volumes (Table 3). During the VFD process, it was observed that vaccine solutions overflowed from the vials when the filling volume exceeded 1.2 mL in 7 mL vials, 1.5 mL in 10 mL vials, and 4 mL in 30 mL vials. Moreover, the virus titer losses of the vaccine preparations that did not overflow ranged from 0.67 ± 0.10 log_10_ to 0.83 ± 0.17 log_10_ when stored at 45℃ for 7 days, and exhibited RMC of no more than 3% (Table 3). Therefore, the optimal filling volumes were determined to be ≤ 1.2 mL in 7 mL vials, ≤ 1.5 mL in 10 mL vials, and ≤ 4 mL in 30 mL vials.

**Table 3:** Effect of fill volume and vial size

Vial (mL)	Vial specification Diameter × Height (mm)	Fill volume (mL)	Liquid level (mm)	Overflow phenomenon ( ±)	NDV titer of products (log EID_50_/mL) (n = 3)		NDV titer loss (log EID_50_/mL)	RM% (n = 8)
					0 days after VFD	Storage at 45℃ for 7 days		
7	22 × 40	0.8	6.5	-	8.50 ± 0.17	7.67 ± 0.17	0.83 ± 0.17	1.99 ± 0.18
		1	7	-	8.65 ± 0.04	7.92 ± 0.12	0.73 ± 0.12	2.01 ± 0.27
		1.2	7.5	-	8.89 ± 0.10	8.06 ± 0.10	0.83 ± 0.10	2.07 ± 0.16
		1.5	9	+	UD	UD	UD	UD
		2	10	+	UD	UD	UD	UD
10	22 × 49	0.8	6.5	-	8.44 ± 0.10	7.67 ± 0.17	0.78 ± 0.17	1.89 ± 0.26
		1	7	-	8.72 ± 0.09	8.06 ± 0.10	0.67 ± 0.10	1.97 ± 0.28
		1.2	7.5	-	8.89 ± 0.10	8.06 ± 0.10	0.83 ± 0.10	2.01 ± 0.27
		1.5	9	-	8.94 ± 0.10	8.17 ± 0.17	0.78 ± 0.17	2.10 ± 0.3
		2	10	+	UD	UD	UD	UD
30	42 × 64	2	6.5	-	8.44 ± 0.10	7.72 ± 0.09	0.72 ± 0.09	1.98 ± 0.23
		3	7.5	-	8.61 ± 0.10	7.89 ± 0.10	0.73 ± 0.10	2.02 ± 0.23
		4	9	-	8.89 ± 0.10	8.06 ± 0.10	0.83 ± 0.10	2.08 ± 0.36
		5	10.5	+	UD	UD	UD	UD

+ : overflow; -: no overflow; UD: undetected. Seventy vials were prepared for each category of filling.

### Optimal shelf temperature for the VFD process at labaratory scale

**Fig. 1:**
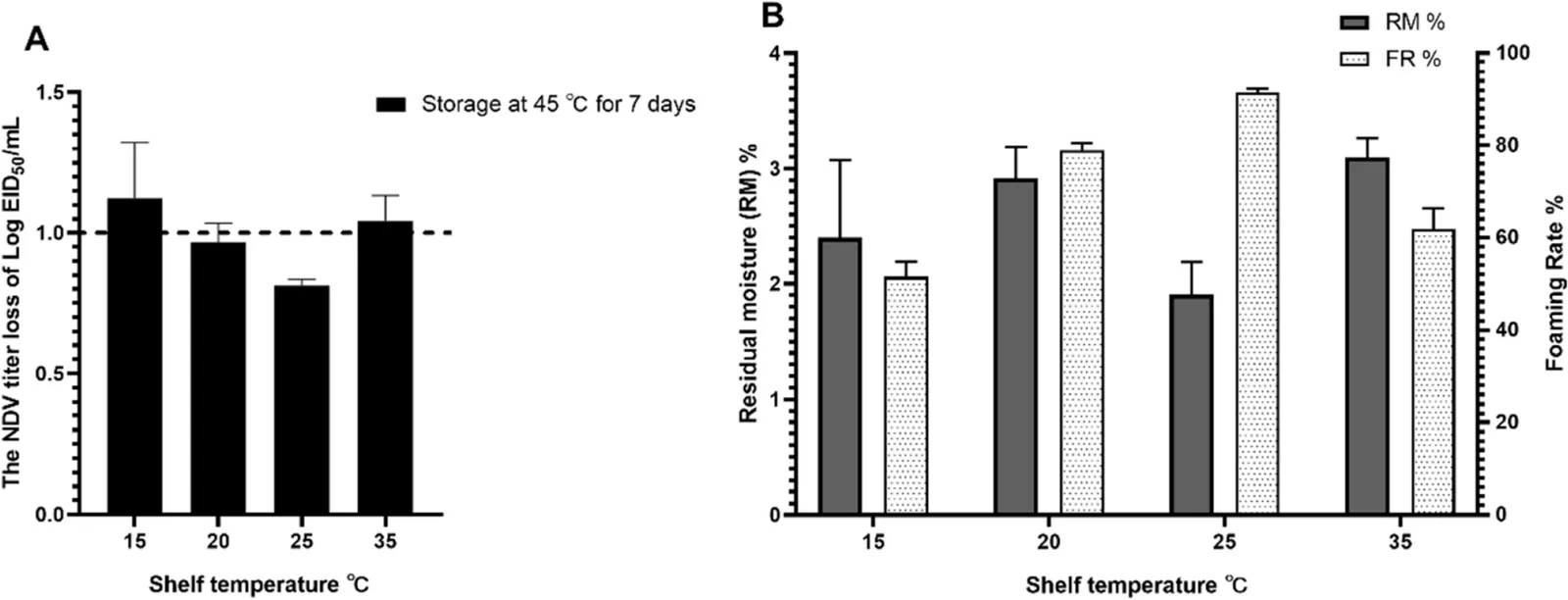
Characteristics of NDV-VFD using 4 different shelf temperatures. The NDV titer loss after 7 days of storage at 45℃ is represented as EID_50_/mL (**A**), the RM% and the FR% (**B**) of VFD vaccines prepared at 4 shelf temperatures

The VFD cycle at laboratory scale was further optimized by determining the optimal shelf temperature and overall drying time. Samples consisting of a 1:1 ratio of virus to T5 formulation were subjected to VFD in the LabLyo1 using the same cycle, with variations in the shelf temperature (i.e. 15℃, 20℃, 25℃, and 35℃)*.* The effect of various temperatures used during the VFD process on the foaming rate, virus titer loss and RMC of the dried samples are shown in Fig. 1. The initial virus titer for all the samples was 8.83 ± 0.07 log EID_50_/mL (not shown). Vaccine preparations with virus titer losses of ≤ 1.0 log_10_, RM% ≤ 3% and FR% ≥ 90% were considered to be efficacious, based on our previously published study (Lyu et al. 2022). These findings indicate that temperatures of 15℃, 20℃ and 35℃ were not conductive to foam formation, as they resulted in products with high residual moisture and poor thermal stability. Furthermore, a lower foaming rate was observed for preparations at these three temperatures compared to the NDV-VFD preparation at a temperature of 25℃ (Fig. 1B). In contrast, preparations at 25℃ showed titer losses of less than 1.0 log_10_, and RM% lower than 3%, corresponding with the highest foaming rate (91.5%) among the temperatures tested. Therefore, the shelf temperature of 25℃was determined to be optimal for the VFD process.

### Determination of optimum overall drying time for the VFD process

**Fig. 2:**
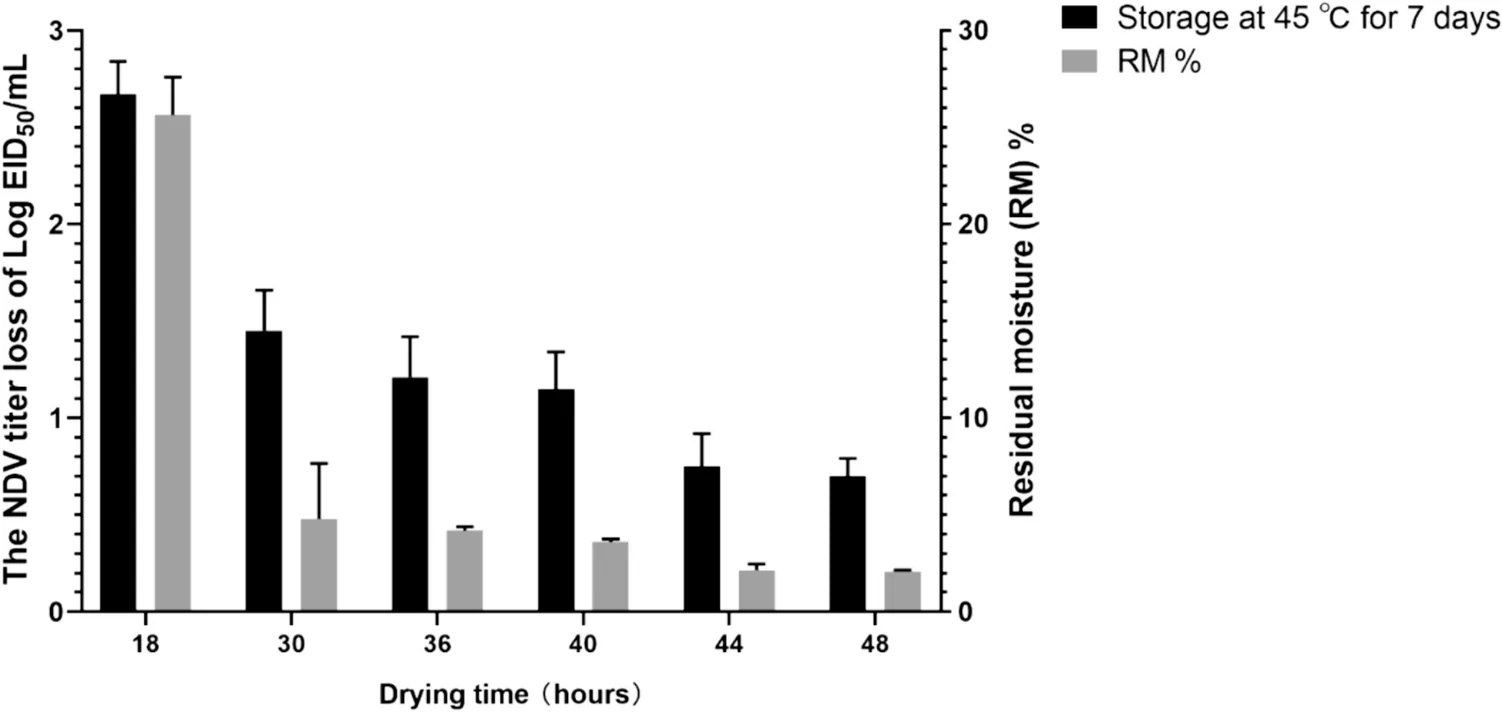
The effect of varying drying times on the NDV titer loss and the RM% of VFD vaccine

With the optimal shelf temperature determined, VFD vaccines were then prepared at different overall drying times (i.e. 18, 30, 36, 40, 44 and 48 h) to determine the optimum drying time for the NDV-VFD process. It was determined that overall cycle drying times of 18, 30, 36 and 40 h were not suitable for NDV-VFD vaccine preparation since the RMC values exceeded 3% (Fig. 2). Notably, samples dried for 18 h exhibited the highest RMC, with an RM% of 25.63%, and a corresponding virus titer loss of 2.67 ± 0.17 log_10_, after storage at 45℃ for 7 days. In contrast, samples prepared using 44 and 48 h drying times exhibited virus titer losses of less than 1.0 log_10_ and RM% < 3%, which were within the acceptable ranges. Therefore, an overall drying time of at least 44 h was determined to be optimal for the NDV-VFD process.

**Fig. 3:**
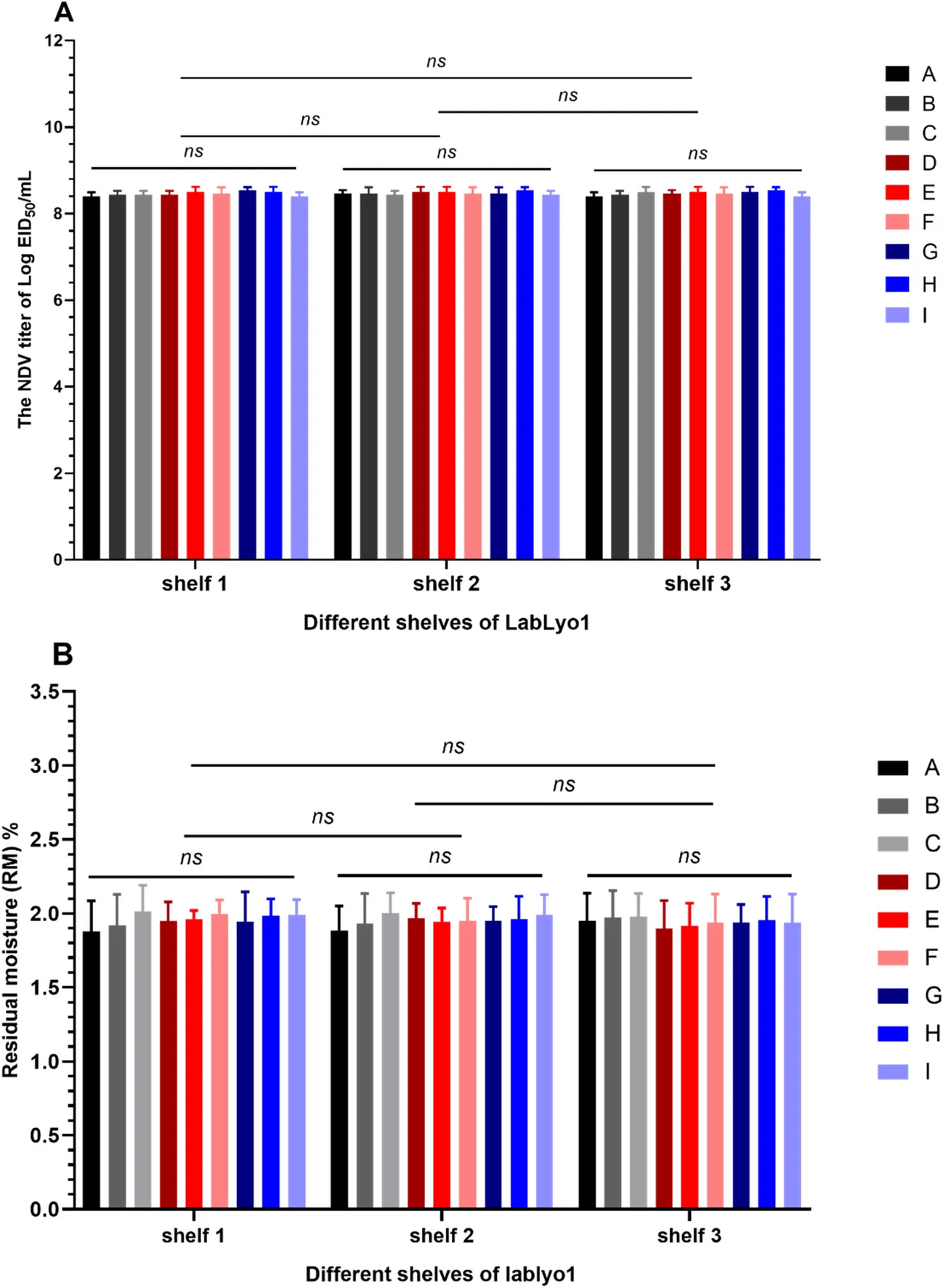
Virus titer loss (**A**) and RM% (**B**) of NDV-VFD preparations in different locations and shelves of LabLyo1 (0.27 m^2^). Vaccines were selected from different positions (**A**-**I**) on shelf 1 to shelf 3 corresponding to A-I in Figure S1. The results are expressed as means ± standard deviation. ^*^, *p* < 0.05; ^**^, *p* < 0.01; ^***^, *p* < 0.005; *ns*, not significant

Further to determining the optimal overall drying time, we next sought to determine if the positioning of the sample during VFD had any effect on the efficacy of the vaccine preparations since samples in the LabLyo1 lyophilizer are located in different positions across three shelves. Therefore, vaccine preparations located on different shelves were selected for AS testing and RM% analysis. As shown in Fig. 3, there was no significant difference (p > 0.05) in the virus titer values of samples taken from different positions and shelves of the lyophilizer, indicating that the VFD process was applied uniformly to all samples in the lyophilizer.

### Thermostability of NDV-VFD vaccine after long-distance transportation without cold-chain

**Fig. 4:**
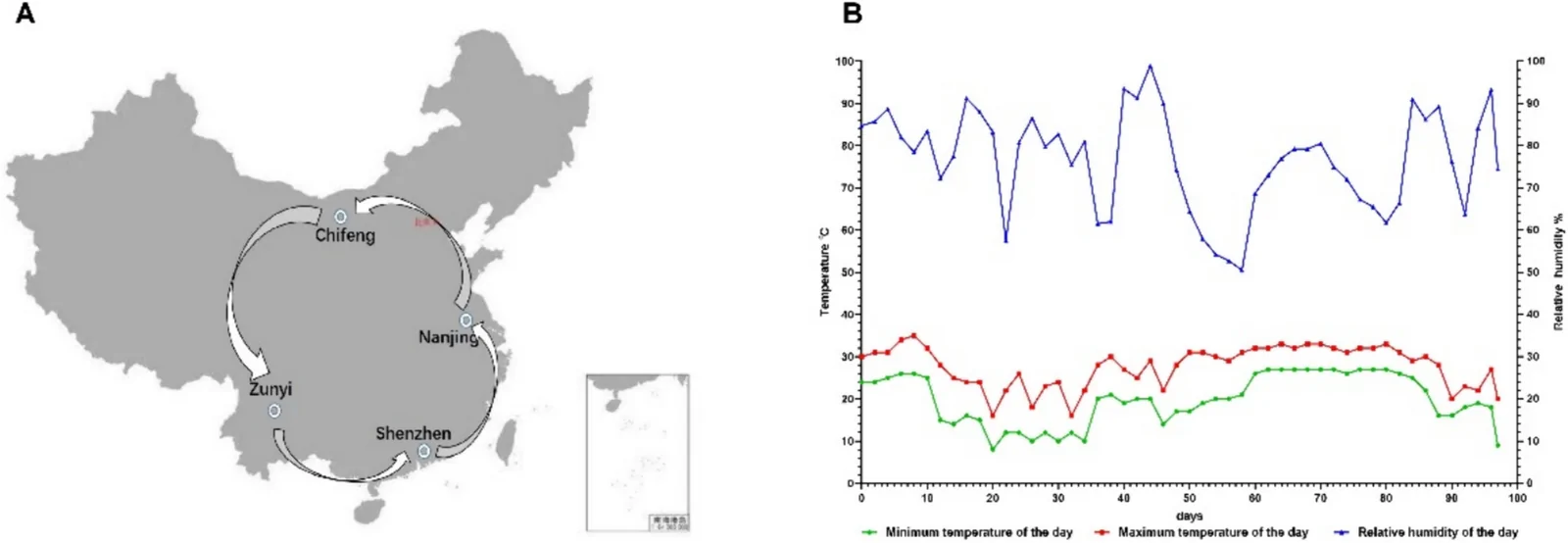
Thermostability of NDV-VFD vaccine preparations after 97 days of transportation in China without cold chain. **A** The route through which the NDV-VFD and the NDV-FD vaccine control samples were transported in China. The samples were transported from (1) Nanjing in Eastern China, to (2) Chifeng in Northern China, to (3) Zunyi in Southwest China, to (4) Shenzhen in South China, and then back to Nanjing. **B** The ambient temperature and humidity during the 97 days of transportation and storage

To evaluate the thermostability of our NDV-VFD vaccine in real life conditions, the live attenuated vaccine preparations were transported around China for 97 days during summer in the absence of cold chain conditions (Fig. 4A). During this time, the highest ambient temperature observed was 35℃, while the lowest temperature recorded was 10℃. Notably, the greatest temperature difference recorded in a single day was 14℃, where the vaccine preparations were subjected to high temperature fluctuation. Similarly, the average relative humidity fluctuated drastically between 50 and 100% (Fig. 4B).

The NDV-VFD vaccine preparations withstood temperature fluctuations (10—35℃) in the absence of cold chain conditions, as evidenced by a low titer loss of 0.56 log_10_ (Table 4). In contrast, the NDV-FD vaccine, which is prepared via freeze-drying, showed higher virus titer losses of 1.61 log_10_ after 97 days of transportation, demonstrating a dependence on cold chain conditions (Table 4). As indicated by these findings, our experimental NDV-VFD vaccine preparation showed improved thermostability compared to the commercially available NDV-FD vaccine.

**Table 4:** The live attenuated NDV vaccines EID_50_/mL before and after long-distance transportation in China

Vaccines	EID_50_/mL of products (log EID_50_/mL) (n = 3)		NDV titer loss (log EID_50_/mL)
	Before transportation	97 days after transportation without cold-chain	
NDV-VFD vaccine	8.56 ± 0.10	8.00 ± 0.17	0.56 ± 0.17
NDV-FD vaccine	8.61 ± 0.10	7.00 ± 0.17	1.61 ± 0.17

### Thermostability of NDV-VFD vaccine from laboratory to pilot scale

The AS test, RM% and foaming rate of the NDV-VFD vaccine from lab-scale to pilot-scale.

Using the NDV-VFD process parameters optimized at laboratory scale, the NDV and T5 formulation (1:1) preparation was vacuum foam dried using lab and pilot lyophilizers to investigate the potential for up-scale application of the process. Subsequently, the vaccine preparations were assessed using the AS test, RM% and FR% for comparison. The results of the AS test showed that virus titer losses of both the lab-scale and pilot-scale preparations after incubation at 45℃ for 7 days did not exceed 1.0 log_10_ (Table 5). Moreover, the RMC of all the lab-scale and pilot-scale NDV-VFD preparations were lower than 3%, with FR% exceeding 90%. Notably, the pilot-scale preparations exhibited the lowest RMC. Collectively, these findings suggest that upscaling the NDV-VFD process from lab-scale to pilot-scale had minimal effect on vaccine efficacy and thermostability.

**Table 5:** The AS test, RM% and FR% of VFD vaccines from lab- to pilot-scale

NDV-VFD vaccines prepared in different scale	Vials	NDV titer of products (log EID_50_/mL) (n = 3)		Virus titer loss after storage at 45℃ for 7 days (log EID_50_/mL) (n = 3)	RM% (n = 8)	FR%
		0 days after VFD	Storage at 45℃ for 7 days			
LabLyo1	400	8.63 ± 0.09	7.84 ± 0.11	0.79 ± 0.04	2.08 ± 0.07	92.3 ± 0.05
LabLyo2	800	8.83 ± 0.12	8.02 ± 0.15	0.81 ± 0.03	2.03 ± 0.03	94.6 ± 0.35
PilotLyo1	5950	8.75 ± 0.07	7.90 ± 0.16	0.85 ± 0.05	1.89 ± 0.18	95.3 ± 0.38
PilotLyo2	11,880	8.87 ± 0.12	7.98 ± 0.11	0.89 ± 0.02	1.84 ± 0.23	95.6 ± 0.55

### The long-term stability of NDV-VFD vaccine at 37℃ from lab-scale to pilot-scale

**Fig. 5:**
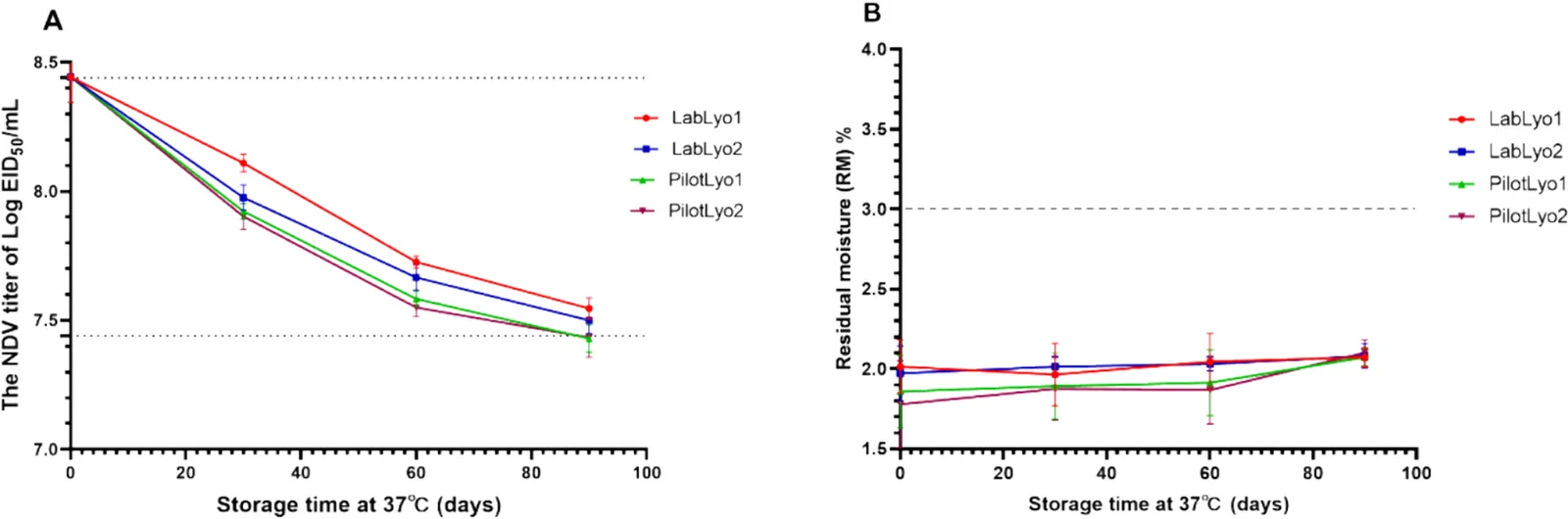
The thermostability and RM% of VFD vaccine stored at 37℃ for 90 days from Lab to Pilot scale. The NDV titer of EID_50_/mL (**A**) and the RM% (**B**) of VFD vaccine stored at 37℃ for 90 days

The long-term stability of the NDV-VFD vaccine preparations produced from lab-scale lyophilizers and pilot-scale lyophilizers were compared by storing the vaccine preparations at 37℃ for 90 days. This temperature is similar to that of hot summer conditions. It was observed that samples prepared using LabLyo1 and LabLyo2 met the prescribed qualifying parameters with virus titer losses of less than l.0 log_10_ and RMC of less than 3% (0.89 ± 0.04 log_10_ and 0.94 ± 0.05 log_10_ respectively). In contrast, samples prepared using PilotLyo1 and PilotLyo2 also showed acceptable RMC of less than 3% (Fig. 5B), but it was observed that the virus titer losses of these samples were approximately l.1 log_10_ (1.04 ± 0.05 log_10_ and 0.99 ± 0.08 log_10_ respectively) (Fig. 5A). These results indicate that there was a slight decrease in the virus infectivity of samples prepared at pilot-scale.

## Discussion

Vaccination remains as one of the most effective measures for prevention and control of diseases, however the efficacy of live attenuated vaccines is usually impacted by thermal inactivation (Singh et al. 2022). Hence, there is a continuous effort to improve the thermostability of live attenuated vaccines. Previous studies showed that using VFD as an alternative to FD improved the thermostability and shelf life of several live attenuated vaccines such as the NDV vaccine, *Salmonella enterica* serovar Typhi-vectored vaccine, rabies vaccine and the vaccine for porcine reproductive and respiratory syndrome (Lv et al. 2016; Lyu et al. 2022; Ohtake et al. 2011; Smith et al. 2015). The improved thermostability of these live attenuated vaccines has been shown through their ability to maintain their efficacy after several months of storage at high temperatures such as 37℃, and even higher temperature (45℃) for as much as 7 days (Litamoi et al. 2005; Lv et al. 2016; Lyu et al. 2022; Ohtake et al. 2011; Smith et al. 2015).

Just like any small-scale process that will be applied to large-scale manufacturing, the VFD process needs to be scalable from lab-scale to pilot-scale. However, upscaling requires further optimization to ensure that product integrity and quality are not compromised during the scale-up process. This study reports the optimization of the NDV vaccine preparation for VFD at laboratory scale as a guide for implementation at pilot-scale. In this regard, we optimized the virus to T5 formulation ratio, liquid filling volume, shelf temperature and overall drying cycle time. All these parameters affect foam formation, residual moisture content, and integrity of the VFD dried product. These factors, in turn, have a significant impact on the infectivity titer and long-term shelf life of the vaccine preparation and subsequently the thermostability of the final product (Bronshtein et al. 2001; Cicerone et al. 2003; Hajare et al. 2010; Lyu et al. 2022; Worrall 2000).

The infectivity titer was determined using the egg infectious dose (EID_50_) method, and the thermostability was considered as the ability of the vaccine preparation to maintain infectivity, as determined by the accelerated aging test (Ghaemmaghamian et al. 2022; Guktur et al. 2021; Osman et al. 2021; Reed and Muench 1938). This was displayed in Fig. 1 where at lower temperature (15℃), the virus titer loss was more than 1.0 log_10_. The loss in the virus titer could be attributed to freezing that was observed in the samples as vapor pressure decreased during the VFD process. At higher temperature (35℃), the virus titer loss and RMC were not within the acceptable range. This may be due to poor foam formation since the solution boiled violently, leading to accelerated water evaporation. Contrastingly, when the shelf temperature was set at ambient temperature (20- 25℃), virus titer loss remained well within the acceptable range of less than 1.0 log_10_. This indicated that the VFD vaccine preparation remained infective even after exposure to the elevated stress conditions (i.e. 45℃ for 7 days). Our data are consistent with previous studies which showed that 25℃ is the optimal temperature during vacuum foam drying process (Lv et al. 2016; Truong-Le 2006).

Various sources of published literature indicate that the overall drying time for different products during the VFD can vary between 10 – 48 h to achieve an acceptable RMC of below 3% (Merivaara et al. 2021; Zhao et al. 2022; Zuo et al. 2020). In the present study, we determined the optimum overall drying time for the NDV vaccine using the VFD process to be at least 44 h. It was consistently observed that shorter overall drying times yielded products with high RMC and reduced infectivity, as indicated by RM% > 3 and virus titer losses greater than 1.0 log_10_. We postulate that the relatively long overall drying time for the NDV vaccine preparation may be attributed to the modest shelf temperature of 25℃, which was maintained throughout the VFD process. Additionally, literature data indicates that the overall drying cycle time for many heat-stable lyophilized vaccines are 48 h or longer, therefore the optimized overall drying time presented in this study can be considered as an improvement to the normal VFD drying time (Bronshein. 2003; Bronshtein et al. 2001; Pisano et al. 2013a; Pisano et al. 2013b; Prabhu et al. 2014; Truong-Le 2006; Walters et al. 2014).

The optimal ratio of NDV to T5 formulation was determined to be 1:1 or 3:2 for the NDV-VFD process. However, of these two ratios, only the ratio of 1:1 was selected for further investigation in this study because NDV-VFD vaccine preparations prepared in a 1:1 ratio of NDV to T5 formulation exhibited the lowest virus titer losses and the lowest RMC. Furthermore, it was established that the maximum liquid filling volumes in vials with different sizes should be kept between 13–17% of the vial capacity to accommodate sample expansion during the VFD process and to prevent sample overflow. This finding was corroborated by previously published data, which indicated that the liquid filling volume of the live attenuated parainfluenza virus (Medi 534) vaccine or monoclonal antibody during VFD should be approximately 15% of the container capacity (Abdul-Fattah et al. 2007a, b).

During lyophilization, there can be discrepancies in product quality, which is influenced by the distribution of the samples throughout the lyophilizer. This technical issue can be attributed to design flaws and mechanical ageing of equipment that cause inconsistent distribution in radiation and heat transfer in the VFD chamber (Pikal et al. 2016; Tsinontides et al. 2004). Therefore, for quality control purposes, it was deemed important in the present study to investigate this factor during NDV-VFD vaccine preparation. We found no significant evidence of any discrepancies in the quality of our product, as the infectivity of our live attenuated NDV-VFD vaccine preparations remained consistent in samples stored on different shelves and positions of the lyophilizer.

The optimized NDV-VFD vaccine preparations were also transported around China for approximately 3 months to assess the thermostability of the vaccine in the uncontrolled real-life scenario. This is critical because after commercialization, the vaccine would be transported regionally or globally, where drastic fluctuations in the ambient temperature and humidity are expected in an unregulated environment. Our data showed that the NDV-VFD vaccine preparations maintained their integrity even after long-distance transportation for around 3 months without cold chain, which was in agreement with the results of the accelerated ageing test in our previous study (Lyu et al. 2022). These findings demonstrate the thermostability of the NDV-VFD vaccine preparation, even in high temperature and high humidity environments. Lastly, it was observed that the optimized NDV-VFD process conditions at lab-scale were transferable to pilot-scale given that the RMC of the dried samples is less than 3%. However, we observed that the virus titer losses for these samples were slightly above the acceptable threshold of 1.0 log_10_. Therefore, further optimization is required for the pilot-scale study to improve the observed virus titer loss.

The aim of this research was to optimize the process parameters for NDV-VFD vaccine preparation and to demonstrate the application of this process at pilot scale. Results from this study showed that the NDV-VFD vaccine prepared at both laboratory and pilot scale can be preserved at 37℃ for 90 days, with RMC not exceeding 3%. Furthermore, the robustness of the NDV-VFD vaccine has been demonstrated in its propensity to withstand long distance transportation without cold chain for about three months. Although further optimization is required for pilot-scale application of the process, this study provide valuable insight for the possible future industrialization of the VFD vaccines.

## Supplementary Information

Below is the link to the electronic supplementary material.

Supplementary file1 (PDF 119 KB)

## Data Availability

The data generated or analyzed during this study are included in this article (and its supplementary information files.)
