# Supplementary material for: Thermostable vacuum foam dried Newcastle disease vaccine: Process optimization and pilot-scale study
Source: Appl Microbiol Biotechnol. 2024 Jun 5;108(1):359. doi: 10.1007/s00253-024-13174-7 (PMC11153293; doi:10.1007/s00253-024-13174-7)
Supplement: Supplementary file 1 — Supplementary file1 (PDF 119 KB) [file 253_2024_13174_MOESM1_ESM.pdf]

Thermostable vacuum foam dried Newcastle disease vaccine: Process optimization and pilot-scale study

Fang Lyu [1,2,4,5], Yan-hong Zhao [1,5], Xiao-xin Zuo [1,6], Babalwa Nyide [2], Bi-hua Deng [1,4,5], Ming-xu Zhou [1, 4], Jibo Hou [1], Jia-jie Jiao [1,5], Min-qian Zeng [1,5], Hong-ying Jie[1], Ademola Olaniran [3], Yu Lu [1,4,5,7*], Thandeka Khoza [2*]

[1] Institute of Veterinary Immunology & Engineering, National Research Center of Engineering and Technology for Veterinary Biologicals, Jiangsu Academy of Agricultural Sciences, Nanjing 210014, China

[2] Department of Biochemistry, School of Life Sciences, College of Agriculture, Engineering & Science, University of KwaZulu-Natal, Pietermaritzburg 3209, South Africa

[3] Department of Microbiology, School of Life Sciences, College of Agriculture, Engineering & Science, University of KwaZulu-Natal, Durban 4000, South Africa

[4] GuoTai (Taizhou) Center of Technology Innovation for Veterinary Biologicals, Taizhou 225300, China

[5] School of Animal Medicine, Nanjing Agricultural University, Nanjing 210095, Jiangsu, China;

[6] Jiangsu Key Laboratory for Food Quality and Safety-State Key Laboratory Cultivation Base, Ministry of Science and Technology, Nanjing 210014, China

[7] School of Pharmacy, Jiangsu University, Zhenjiang, 212013, China

*****Correspondence:

khozat1@ukzn.ac.za; Tel: +27 33 2606281

luyu@jaas.ac.cn (Y. Lu); Tel: +86-25-8439-2088

Supplementary information

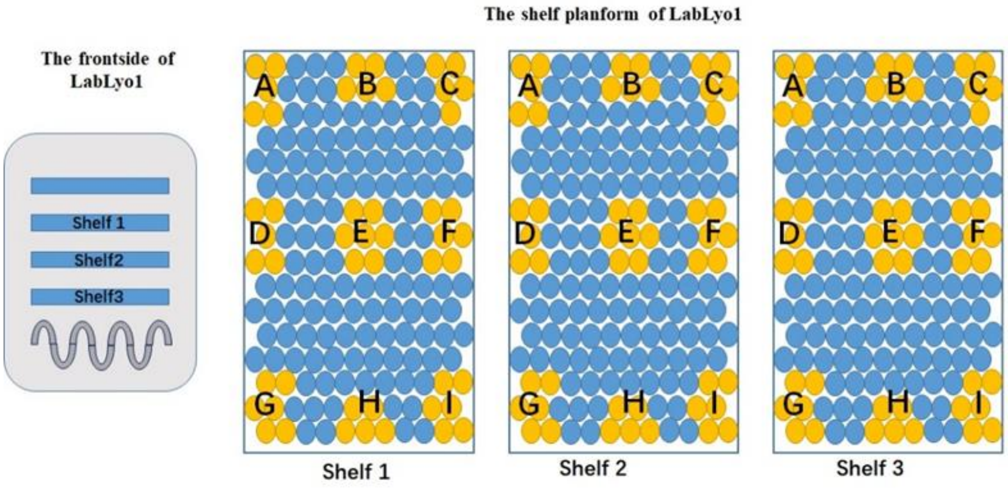

Figure S1 The sampling design showing different positions of the selected NDV-VFD vaccine samples in the shelves. There are 3 shelves in the lab lyophilizer (0.27 m^2). The colors represent the location and position of the samples that were selected for analysis.
